# Reduced mammillary body volume in individuals with a schizophrenia diagnosis: an analysis of the COBRE data set

**DOI:** 10.1038/s41537-023-00376-7

**Published:** 2023-08-01

**Authors:** Michal M. Milczarek, Syed Irtiza A. Gilani, Maarten H. Lequin, Seralynne D. Vann

**Affiliations:** 1 School of Psychology, Cardiff University, Tower Building, Cardiff, CF10 3AT UK; 2 Neuroscience and Mental Health Innovation Institute, Hadyn Ellis Building, Cathays, Cardiff, CF24 4HQ UK; 3 CUBRIC, School of Psychology, Maindy Road, Cardiff, CF24 4HQ UK; 4 Division Imaging & Oncology, Department of Radiology & Nuclear Medicine, University Medical Center Utrecht & Princess Máxima Center for Pediatric Oncology, 3508 GA Utrecht, The Netherlands

**Keywords:** Schizophrenia, Neural circuits

## Abstract

While the frontal cortices and medial temporal lobe are well associated with schizophrenia, the involvement of wider limbic areas is less clear. The mammillary bodies are important for both complex memory formation and anxiety and are implicated in several neurological disorders that present with memory impairments. However, little is known about their role in schizophrenia. Post-mortem studies have reported a loss of neurons in the mammillary bodies but there are also reports of increased mammillary body volume. The findings from in vivo MRI studies have also been mixed, but studies have typically only involved small sample sizes. To address this, we acquired mammillary body volumes from the open-source COBRE dataset, where we were able to manually measure the mammillary bodies in 72 individuals with a schizophrenia diagnosis and 74 controls. Participant age ranged from 18 to 65. We found the mammillary bodies to be smaller in the patient group, across both hemispheres, after accounting for the effects of total brain volume and gender. Hippocampal volumes, but not subiculum or total grey matter volumes, were also significantly lower in patients. Given the importance of the mammillary bodies for both memory and anxiety, this atrophy could contribute to the symptomology in schizophrenia.

## Introduction

Schizophrenia constitutes only a minor proportion of all psychiatric disease cases, however, given the severity and range of associated symptoms, its impact on affected individuals and their families is especially high. Schizophrenia episodes are typically associated with hallucinations and thought disturbances, but individuals are also affected by difficulties in perception, executive function, and other cognitive skills, including memory and emotional processing. While there are treatments for the positive symptoms, there is very little available to alleviate chronic cognitive impairments, which contribute significantly to the long-term impact on patients and their families. Schizophrenia research has focused in large part on the prefrontal cortex, striatum and especially, the hippocampus^1^. However, it is becoming clear that far more widespread circuits are implicated, contributing to the complexity of the disorder. A better understanding of the circuitry affected in schizophrenia will help improve treatment options.

The mammillary bodies form part of the medial diencephalon and play an important role in the integration of positional, sensory information and arousal, which underlies the formation of complex memories, i.e., spatial, contextual, and temporal memories^2^. The mammillary bodies receive dense inputs from the hippocampal formation, via the fornix, and from Gudden’s tegmental nuclei. They project, in turn, to the anterior thalamic nuclei via the mammillothalamic tract, and to Gudden’s tegmental nuclei via the mammillotegmental tract. The mammillary bodies support the formation of memories by co-ordinating oscillatory activity across hippocampo-cortical regions^3^. Damage to the mammillary bodies, and their projections via the mammillothalamic tract, result in episodic and recollective memory impairments in patients and spatial memory impairments in animal models^4^. Mammillary body pathology has been reported in multiple neurological disorders^5–7^, however, the integrity of the mammillary bodies in schizophrenia is not currently clear. Several post-mortem studies have assessed the status of the mammillary bodies in individuals with schizophrenia. One study found reduced neuronal density along with enlarged volume in patients with schizophrenia, resulting in no net change in overall neuronal number^8^. However, data in the patient group in this study exhibited higher variance than in controls and the authors did not provide an age range for the participants. Another post-mortem study found a marked loss of parvalbumin neurons in the mammillary bodies^9^. In both cases, changes were more pronounced in the left hemisphere than the right^8,9^. However, since post-mortem studies are often limited to relatively small samples—particularly for the mammillary bodies which are more likely to be damaged on extraction given their location—it has been challenging to relate the observed changes to ageing and other stratifying factors. Furthermore, the mammillary bodies are particularly sensitive to hypoxia, so cell loss may be harder to interpret in post-mortem studies as they can be affected by the cause of death^10,11^.

Analysing mammillary body volumes from in vivo MRI studies can address some of the limitations with post-mortem studies. One MRI study that assessed mammillary body volume in patients with a schizophrenia diagnosis found a trend for the right mammillary body to be larger in patients with schizophrenia compared to controls^12^. A further study used a larger database to assess mammillary body volume in patients with schizophrenia and found no difference^13^. However, the inter-rater correlation was quite low for the mammillary bodies, and lower than for other structures in the study, which may have affected the accuracy of mammillary body delineation^13^. Reduced fornix integrity has also been reported in patients with schizophrenia^14^; however, the fibres that innervate the mammillary bodies constitute only part of the entire fornix, as such it is not clear whether these fornical changes relate to mammillary body pathology.

Therefore, based on current studies, the nature of mammillary body pathology in schizophrenia remains unclear. This is likely due to multiple factors, including the heterogeneity of schizophrenia, stratification of the patient population and differences in imaging protocols and/or the segmentation of the mammillary bodies. To address some of these issues, we carried out a volumetric analysis of an open-source data set with 72 participants with a schizophrenia diagnosis and 74 age-matched controls (COBRE, see Methods below). Due to the small size of the mammillary bodies and their proximity to the third ventricle (whose size may be affected in schizophrenia), mammillary body volumes were segmented manually by an experienced researcher. Three additional automatically extracted volumes were included in the analyses: the hippocampus, the subiculum, and the total grey matter. The hippocampal formation has been repeatedly shown to be affected in schizophrenia e.g. ref. ^15^, and it constitutes the primary excitatory input to the mammillary bodies, mainly via the subiculum. Mammillary body atrophy can arise from anterograde degeneration following hippocampal damage, therefore, including hippocampal volumes would allow us to determine whether any mammillary body changes might be driven by hippocampal volume loss. Given the hippocampal projections to the mammillary bodies predominantly arise from the subiculum, this subregion was included separately^16^. Inclusion of total grey matter enabled contrasting regional changes with whole-brain atrophy. To further account for inter-individual differences, we also performed analyses following normalisations to total brain volume and gender, using three different commonly used methods: a ratio method, a covariance method and an allometric method to account for non-linearity in the scaling of sub-cortical regions^17^ (see ‘Methods’ for further details). Finally, we also investigated the effect of age on regional volumes.

## Materials and methods

### COBRE database

The Center for Biomedical Research Excellence (COBRE) contributed a total of 146 structural MR images data from 72 patients with a schizophrenia diagnosis and 74 healthy controls. In the control group there were 23 females (age 18–58) and 51 males (age 18–65; one left-handed). In the patient group there were 14 females (age 20–65; one left-handed) and 58 males (age 18–64; 9 left-handed). Participants had been excluded from the study if they had any neurological disorders, severe head trauma with more than 5 min loss of consciousness, history of substance abuse or dependence within the last 12 months. Diagnostic information for this database was collected using the Structured Clinical Interview used for DSM Disorders (SCID). The majority of the patients were classified with paranoid type (*n* = 42) with the next highest classification being residual type (*n* = 12). The remaining patients were distributed across disorganised (*n* = 3), catatonic (*n* = 2), schizoaffective disorder (*n* = 5), undifferentiated (*n* = 6) and not specified (*n* = 2).

### MRI acquisition

All images were collected on a 3 T Siemens Trio scanner. A sagittal five-echo 3D MPRAGE (Multi-Echo MPR) sequence was used with the following parameters: TE_1_ = 1.64 ms, TE_2_ = 3.5 ms, TE_3_ = 5.36 ms, TE_4_ = 7.22 ms, TE_5_ = 9.08 ms, TR/TI = 2530/900 ms, Flip angle = 7°, FOV = 256 × 256 mm, Slab thickness = 176 mm, Matrix = 256×256×176, Series = interleaved, Multislice mode = single shot, Voxel size = 1×1×1 mm, Pixel bandwidth = 650 Hz. The total scan time was 6 min. TR, TI and time to encode partitions for this acquisition method are similar to that of a conventional MPRAGE, however, with 5 echoes it resulted in visually better grey matter–white matter–cerebrospinal fluid contrast compared to typical MPRAGE.

The imaging data and phenotypic information for COBRE database was collected and shared by the Mind Research Network and the University of New Mexico funded by a National Institute of Health Center of Biomedical Research Excellence (COBRE) grant 1P20RR021938-01A2.

### Mammillary body volumes

All structural 3D volumes were re-sampled on a 0.5 × 0.5 × 0.5 mm grid prior to any volumetric analyses using FSL (FMRIB Software Library) to improve resolution and accuracy. The left and right mammillary bodies were segmented manually using the ITK-SNAP software [version 3.8; www.itksnap.org^18^] and volumes were obtained using the same software.

**Fig. 1:**
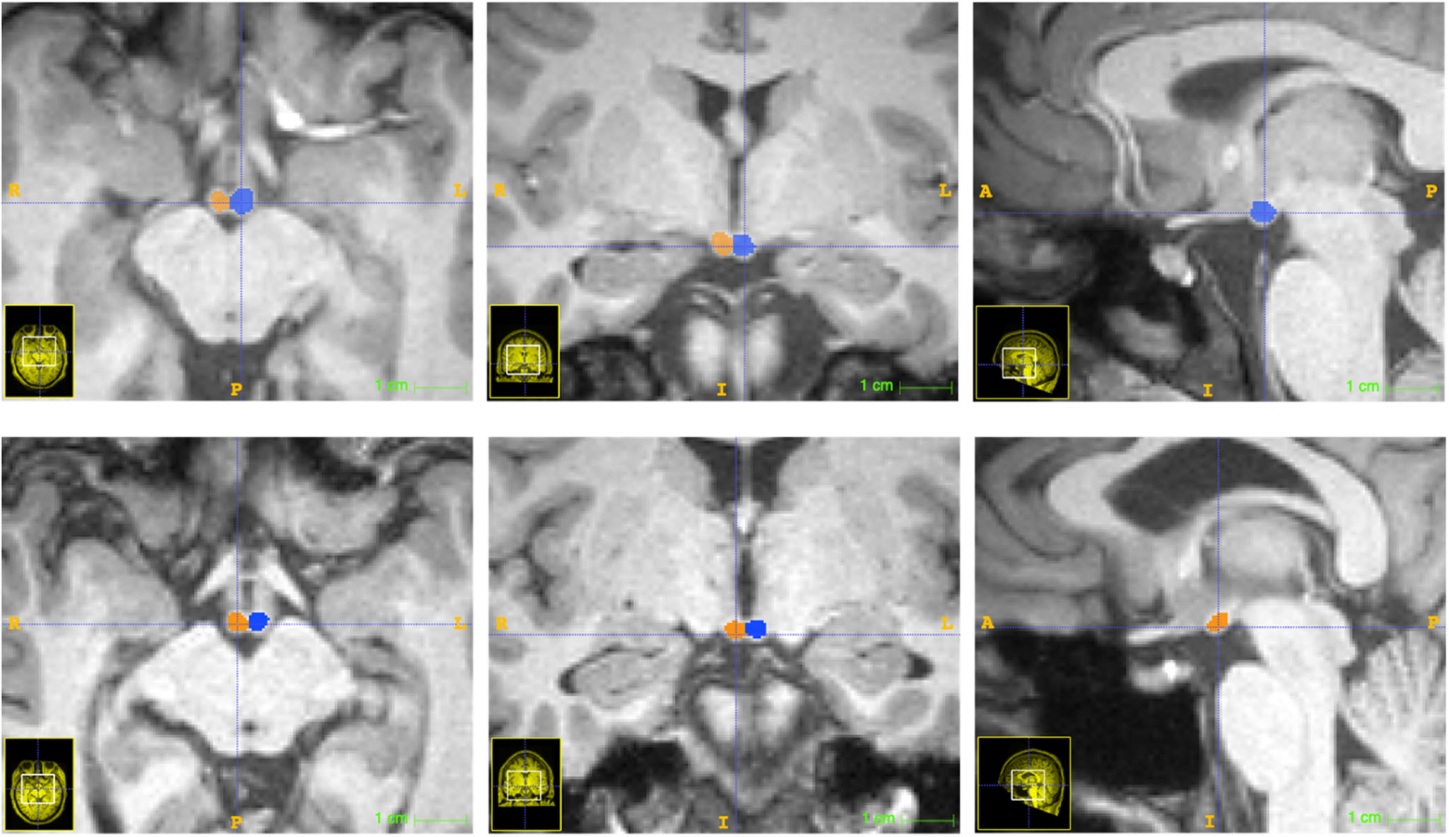
Two examples of mammillary body segmentation using the ITK-SNAP software. Each image row represents axial, coronal and sagittal images from the same brain, respectively.

The mammillary bodies are spherical structures at the base of the brain. The demarcation of mammillary body boundaries was guided by its fibre encapsulation across three different planes (Fig. 1). The inferior boundaries of the mammillary bodies were identified by CSF signal intensity from the interpeduncular fossa. The lateral boundaries were identified from the border with the substantia nigra. On axial sections, the posterior boundaries were identified by the interpeduncular fossa while the third ventricle was used for the anterior boundary. On sagittal images, the mammillary bodies are clearly seen to protrude ventrally and the surrounding CSF was used to identify the more ventral boundaries, while the fibre encapsulation and origin of the mammillothalamic tract was used to identify its more dorsal boundaries^19^. All segmentations were carried out without knowledge of the demographic data or group allocations. The same experimenter (S.D.V.) carried out all segmentations; repeat segmentations were carried out on a subset of scans to acquire an intra-class correlation coefficient as a measure of reliability. Segmentations were visually inspected by a second experimenter (S.G.) for quality control. Again this was done without knowledge of group allocation (see Supplementary Information for further examples). Three measures were obtained for each participant: MB_L, MB_R and MB_T corresponding to the left, right and total mammillary bodies.

### Whole brain volumes

FreeSurfer^20^ version 7.3 was used with default settings for evaluating total brain volume (Brain Segmentation Volume Without Ventricles), intracranial volume (see Supplementary Information) and total grey matter volume using the 3D T_1_-weighted MRI scans.

### Hippocampal volumes

Hippocampal volumes were measured using the FreeSurfer’s subregion segmentation utility provided with FreeSurfer 7.3. The left and right hippocampus volumes were calculated separately for the whole hippocampus, whole hippocampal body, whole hippocampal head, subiculum body and subiculum head subregions. Following extraction of the subregions, they were merged into whole hippocampus (HPC_L, HPC_R) and whole subiculum (SUB_L, SUB_R) for each hemisphere as well as total volumes (HPC_T, SUB_T).

### Statistical analysis

Statistical analysis and data handling were performed in Matlab 2022b (Mathworks, USA) using built-in functions unless otherwise stated.

#### Demographic statistics

Ages were compared across groups using a *t* test while a Chi-squared test was used to compare gender and handedness distributions across groups.

#### Volumetric comparisons

We utilised a generalised linear model (GLM) approach with each subregion modelled separately. Data were analysed for Raw (unadjusted) volumes and following three normalisation procedures to adjust for total brain volume (TBV): the Ratio, Covariance and Allometric techniques^17^. The Ratio method involved dividing regional volumes by the TBV for each participant; the Covariance method involved regressing out the effect of TBV so that residuals could be compared and lastly, the Allometric method was analogous to the Covariance method except it used log10 transformed data. The distributions of residuals for each model were visually assessed using Q-Q plots and accepted as approximately normal. The effect of Gender was accounted for where it significantly improved model fits: the most parsimonious model was chosen for each region based on a log likelihood ratio test comparing a model with an interaction term, an additive model and a simple (no Gender as a factor) model. For raw and ratio adjusted volumes, Gender was factored in when testing for the effect of Group whereas for covariance and allometrically adjusted volumes, it was factored in during the normalisation process and then again when testing for the Group effect. The effect of Age was probed in covariance and allometrically adjusted volumes (as Gender was already accounted for in these datasets) following the same iterative model comparison method as described above. Data were also fitted with a segmented regression approach (using the SegReg software; https://www.waterlog.info/segreg.htm)—to test whether the rate of volume change differed with age—and split into younger and older cases based on the presence of a significant regression breakpoint.

Since data for some region × condition combinations were not normally distributed, we utilised medians and interquartile ranges as measures of central tendency and variability, respectively. Group differences, where significant, were reported along with median-based effect sizes (Cohen’s *d* calculated with median and median absolute deviation). All *p* values were subjected to family-wise multiple comparison correction (Bonferroni method). Data were plotted following *z*-score transformations (to allow for simultaneous visualisation of vastly different volumes and units, using a median-based, ‘robust’ normalisation method) with gramm^21^ in Matlab and modified in PowerPoint (Microsoft Office, USA). An *α* threshold of 0.05 was selected for significance.

## Results

### Demographics

Table 1 displays summary statistics for Age, Gender and Handedness for both groups. The proportion of left-handed individuals was higher in the Patient group (*χ*^2^ = 6.3, *p* = 0.012), while Age and Gender did not differ between groups. Since only a very small proportion of individuals displayed left-handedness (<7%), this measure was not included in subsequent analyses.

**Table 1:** Demographic data.

Index	Controls (*n* = 74)	Patients (*n* = 72)	*t* value/*χ*^2^	*p* value
Age	35.82 ± 11.58	38.17 ± 13.89	−1.11	0.27
Gender (F:M)	23:51	14:58	2.61	0.11
Handedness (L:R:B)	1:71:2	9:61:2	6.3	0.012

Age is mean ± standard deviation. *T*-values are reported for age and *χ*^2^ for gender and handedness. L left handed, R right-handed, B use both left and right hands.

*L* left handed, *R* right-handed, *B* use both left and right hands.

#### Volumetric differences

We analysed estimated volumes for the mammillary bodies, the hippocampus and the subiculum, as well as total grey matter. All regions other than the mammillary bodies were automatically extracted while the mammillary bodies were manually segmented. The intra-class correlation coefficient for manual segmentation was high (0.94). The scatterplots for the unadjusted mammillary body volumes are provided in the Supplementary Information (Figs. S1 and S2).

We utilised unadjusted (Raw) as well as Total Brain Volume-normalised volumes derived using three different methods (Ratio, Covariance, Allometric) to ensure the robustness of the analyses. The effect of Gender was accounted for where it significantly improved the model fit (see ‘Methods’).

**Fig. 2:**
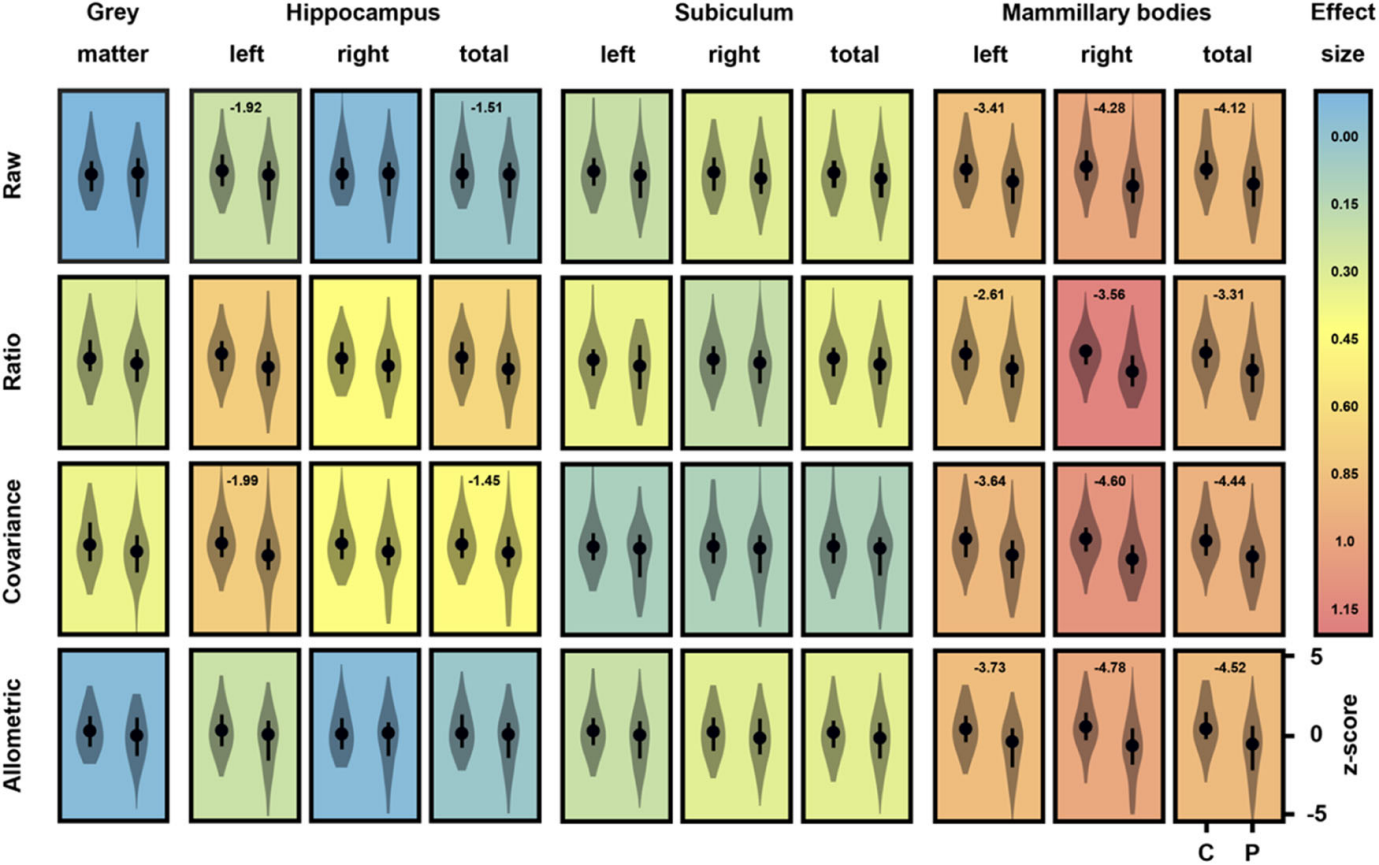
Volumetric differences. The composite chart displays violin plots of z-scored regional brain volumes in Controls (C) and Patients (P) either unadjusted (Raw) or normalised by Total Brain Volume (and Gender) according to the Ratio, Covariance and Allometric techniques (see ‘Methods’). The dots represent median values and error bars, the interquartile range. The colour of the background in each plot indicates the effect size of the difference (median-based Cohen’s *d*) while the numbers on top of the plots are log10 *p* values for significant differences only.

Using Raw volumes, significant differences were found in the mammillary bodies (*t*_left_ = −4.25, *p*_left_ = 3.9 × 10^−4^; *t*_right_ = -4.7, *p*_right_ = 5.3 × 10^−5^; *t*_total_ = −4.64, *p*_total_ = 7.6 × 10^−5^) as well as in the hippocampus (*t*_left_ = −1.99, *p*_left_ = 0.01; *t*_total_ = −3.01, *p*_total_ = 0.03), revealing lower volumes in patients with schizophrenia. Normalisation methods preserved the main effect of Group in the mammillary bodies and, for the covariance method, in the hippocampus (see Fig. 2 for details). Whereas the hippocampus showed differences only in the left hemisphere (or when analysed as a whole), mammillary body volumes displayed the same degree of volume loss across both hemispheres (allometric method: *z* = 0.1, *p* > 0.05, z-test)^22^. A similar pattern of findings was observed when intracranial volume was used to control for grey matter volume (Table S1).

#### Relationship between mammillary body and hippocampal volumes

**Fig. 3:**
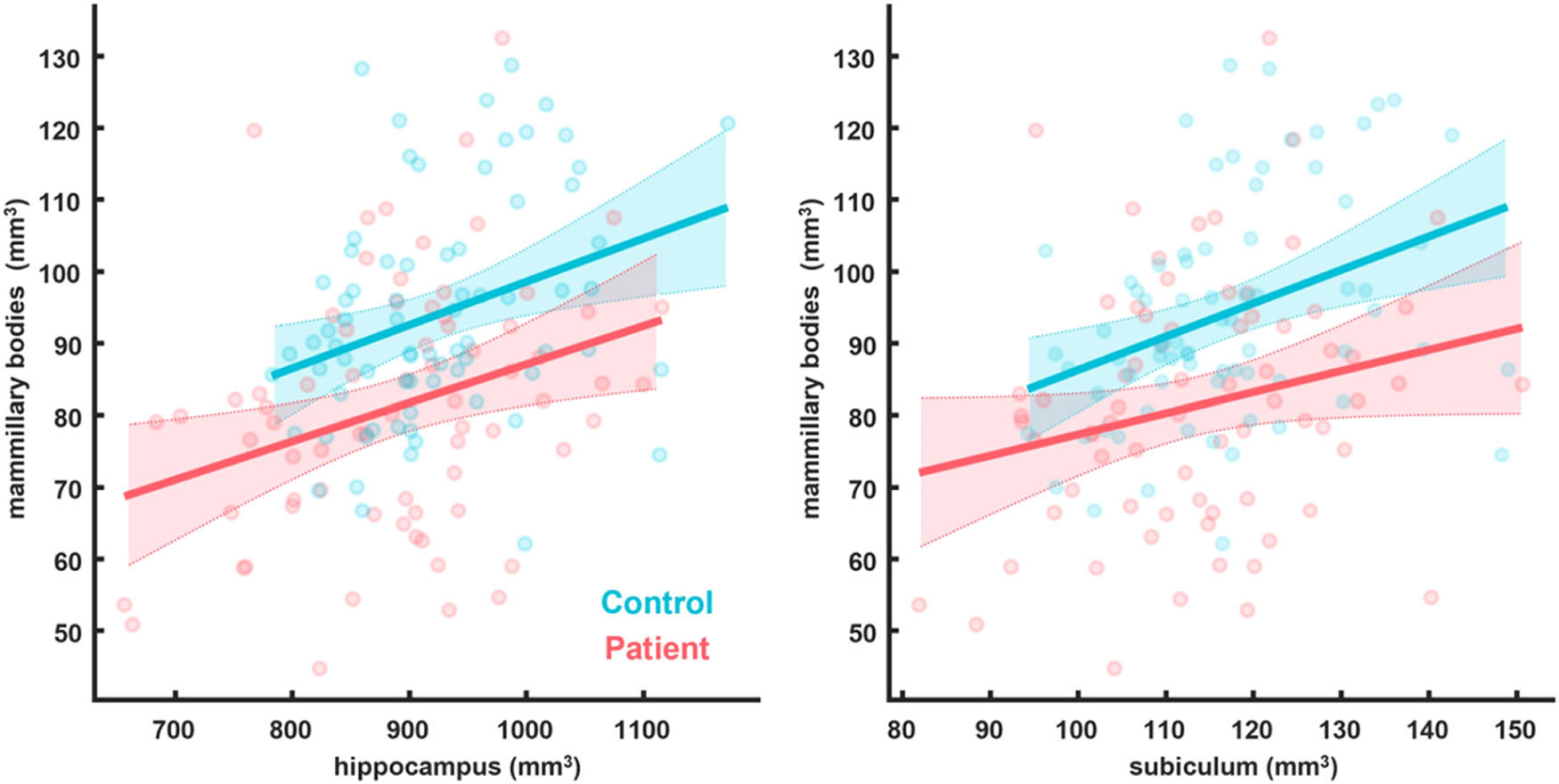
Scatterplots of the relationships between unadjusted total mammillary body volume and total hippocampal and subicular volumes in Controls and Patients. The lines are general linear model fits and the shaded areas, the 95% confidence intervals.

When looking at the relationship between mammillary body and hippocampal volumes (both unadjusted and normalised), the general trend is for a moderate but significant correlation between the total hippocampus and mammillary body volumes, for both Patients and Controls (Table 2 and Fig. 3). For the relationship between subiculum and mammillary body volumes, there is a moderate correlation for the Control group but not for the Patient group (Table 2 and Fig. 3).

**Table 2:** The relationship between total mammillary body volumes and total hippocampal and total subiculum volumes.

Correlation	Group	Raw	Ratio	Covariance	Allometric
MB-SUB	Control	0.36 (−2.23)*	0.27 (−1.08)	0.2 (−0.44)	0.37 (−2.32)*
	Patient	0.23 (−0.64)	0.22 (−0.57)	0.06 (0)	0.25 (−0.84)
MB-HPC	Control	0.32 (−1.66)*	0.2 (−0.46)	0.11 (0)	0.31 (−1.55)*
	Patient	0.31 (−1.52)*	0.3 (−1.38)*	0.17 (−0.21)	0.33 (−1.74)*

The values represent Pearson correlation coefficients and associated, Bonferroni-adjusted (log10) *p* values in brackets. *Significant relationships.

#### Relationship with Age

**Fig. 4:**
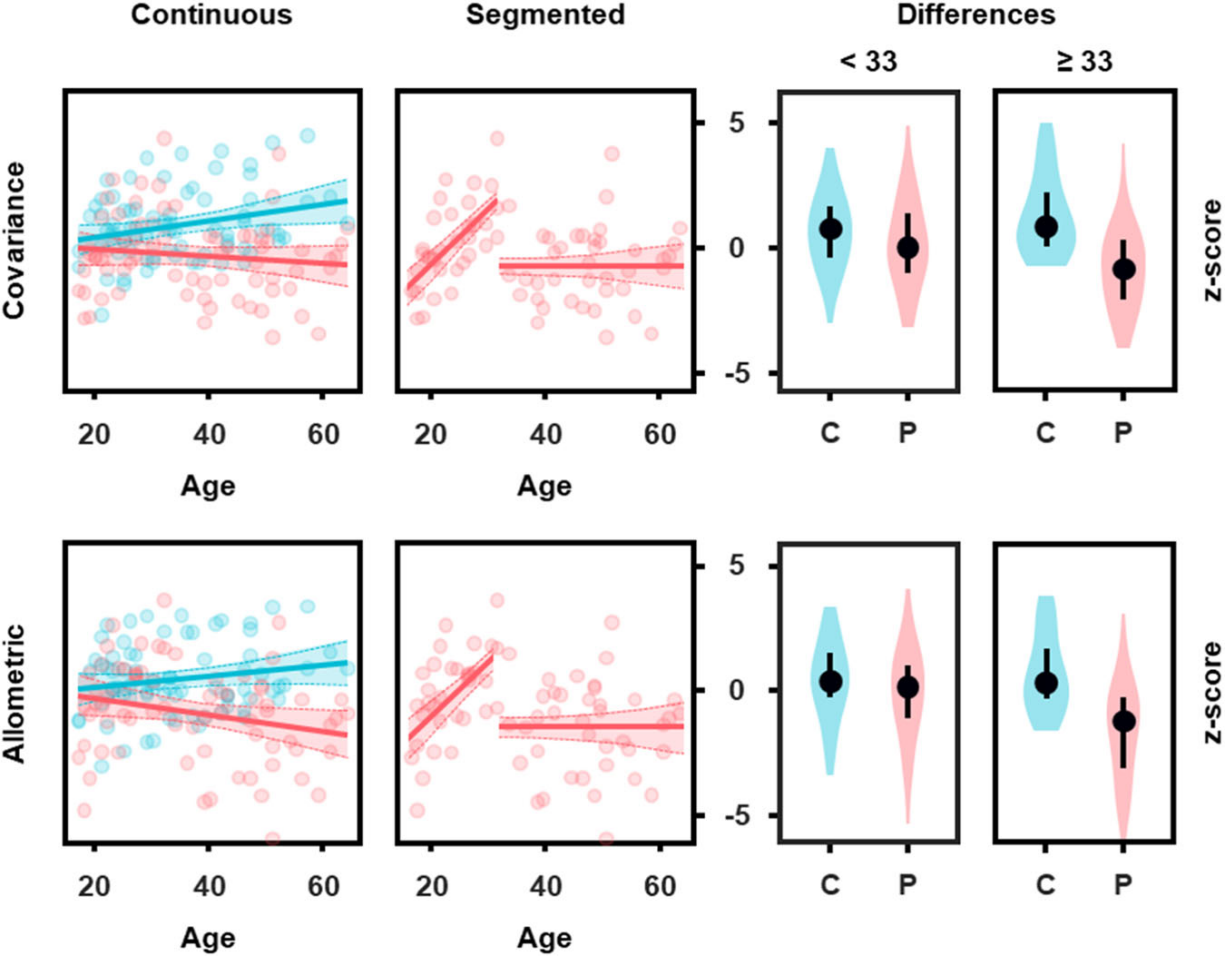
Relationship of mammillary body volume with age. The scatterplots on the left display fits for covariance and allometrically adjusted mammillary body volumes versus Age modelled with continuous regressions, in patients and controls, as well as segmented regressions in patients only (there was no breakpoint for controls). The shaded areas represent 95% confidence intervals. On the right, the violin plots display data split according to the segmented regression breakpoint at 33 years of age. The dots are medians and error bars, the interquartile range.

We further investigated whether the observed volumetric differences in (total) mammillary body volume were age dependent. Based on both covariance and allometrically adjusted volumes, there was a significant Age by Group interaction explaining 16% of the observed variance (*t* = −2.35/−2.50, *p* = 0.02/0.02 for the covariance and allometric methods, respectively), revealing age-related mammillary body atrophy in Patients with schizophrenia (at a rate of −4.26% per decade). Since the relationship between Age and mammillary body volume in Patients appeared non-linear (Fig. 4), we sought to model it with a segmented regression approach (SegReg; https://www.waterlog.info/segreg.htm). Indeed, the rate of volume change differed by Age, as evidenced by the presence of a significant breakpoint (covariance: *F*(3,67) = 7.6, *p* < 1.9 × 10^−4^, allometric: *F*(3,67) = 5.2, *p* < 1.9 × 10^−3^). In younger Patients (<33.04 years old), there was a positive relationship between volume and Age (covariance: *t* = 4.67, *p* = 5.49 × 10^−5^, allometric: *t* = 3.79, *p* = 6.54 × 10^−4^) while older Patients (≥33.04 years old) did not display any relationship with Age (Fig. 2). Control cases did not exhibit a significant breakpoint but a small, continuously positive relationship with Age (covariance method only: *t* = 2.33, *p* = 0.02). Consistent with this, data split according to the breakpoint revealed volume reductions only in older patients with schizophrenia (*t* = −5.66/−5.66, *p* = 2.56 × 10^−7^/2.58 × 10^−7^ for the covariance and allometric methods, respectively), explaining 29% of the observed variance.

## Discussion

Schizophrenia is a complex disorder, and the underlying neural basis is still uncertain. Limbic brain regions have been repeatedly implicated but the findings for the mammillary bodies have been mixed. We made use of a cross-sectional sample of 146 participants to improve power and to enable us to study the effects of age, gender, and brain hemisphere on mammillary body volume. The results provide evidence for schizophrenia-mediated atrophy of the mammillary bodies (as well as the hippocampus), which is robust against age-related and inter-individual differences in whole-brain volume. While overall grey matter typically decreases at a greater rate in patients with schizophrenia than in controls^23^, the mammillary body volume loss we report exceeded that explained by grey matter decline alone.

As with previous studies, the left mammillary body was found to be slightly larger (6.4%) than the right in control participants^12,24^. In the current data set, the left mammillary body was also larger (6.96%), than the right in the patient group, whereas a previous study reported the left and right mammillary bodies to be of equivalent volumes in patients with schizophrenia^12^; a further study did not separate mammillary body volumes by hemisphere^13^. While there were hemispheric differences in mammillary body volume in both the patients and controls in the present data set, the hemispheres were not differentially affected in schizophrenia with an equivalent reduction in volume in both the left and the right hemisphere. While other studies have reported increases in volume on either the right^12^ or the left^8^ hemisphere, consistent with the present study, one post-mortem study found bilateral mammillary body neuronal loss, although the loss was larger on the left^9^. A recent study also found a reduction in the dopamine D3 receptor - a receptor implicated in the development and treatment of schizophrenia^25^—in both left and right mammillary bodies of patients with schizophrenia^26^. Together with the present data, this suggests that the mammillary bodies are impacted across both hemispheres in schizophrenia.

There were no significant interactions between mammillary body volume and gender across the patient and control groups. However, numerically, there was a greater volume decrease in male patients (15.5%) compared to female patients (9.5%). As there were more males than females in the present data set, it is possible that with balanced groups, this difference would become significant. Previous studies either did not look at effects of gender^12^ or found no effect of gender^13^. Determining whether the mammillary bodies in men are more vulnerable in schizophrenia, or whether the numerical differences found in the present study might be explained by differences in additional lifestyle factors, is an important future goal.

The current findings differ from previous MRI studies that either reported no difference in mammillary body volume^13^ or an increase, driven by changes in the right hemisphere^12^. These differences may be due to the boundaries used for mammillary body segmentation and the extent to which adjacent hypothalamic nuclei and/or white matter were included in the measurements. It also could be due to the present study using all planes, rather than just the coronal plane, for segmentation, as well as re-sampling images, which may improve accuracy. The smaller sample size (26 patients versus 26 controls) in the Tognin et al. study^12^ could also contribute to the differing results given variation in mammillary body volumes, even in control populations. In the current dataset, there was also variability in the patient group with some patients showing little or no mammillary body atrophy and others showing very marked volume loss. This is consistent with schizophrenia being highly heterogenous and likely encompassing different subcategories, some of which may be more predisposed to mammillary body pathology. Furthermore, there appears to be a complex relationship with age, which is likely driven by disease stage and could explain some of the previous contrasting findings depending on the age of participants. In younger patients there was a sharp rise in mammillary body volume, which may be consistent whith initial inflammation and swelling during early disease stages and the reported volume increase in animal, post-mortem and MRI studies^8,12,27^, followed by subsequent atrophy during later disease stages. Non-linear changes have previously been reported in longitudinal studies with more dynamic temporal patterns of mammillary volumes, including volumetric increases at the earliest stages of illness^28^. Large-scale longitudinal studies are needed to better understand these variations across patients and age and how they relate to disease progression and symptomology. Furthermore, it would be important to determine whether early markers of mammillary body pathology could be used to better stratify patients for subsequent treatment and neuroprotective protocols and whether there are potential changes within the mammillary bodies prior to symptom onset.

The mammillary bodies receive a dense input from the hippocampal formation via the fornix^2^; given both the hippocampus and fornix have been implicated in schizophrenia, one possibility is that mammillary body atrophy reflects the loss of hippocampal inputs. We obtained automatic estimates of hippocampal and subiculum volumes and found a modest reduction in the hippocampal volumes but no significant changes for the subiculum. Furthermore, for the patient group there were only moderate relationships between hippocampal and mammillary body volumes and no significant correlations between mammillary body and subiculum volumes, again suggesting the mammillary body volumes were not being directly driven by hippocampal/subiculum atrophy. The reduction in hippocampal volume was consistent with the unadjusted volumes previously reported for this same data set [left and right hippocampus reduced in patients by 4% and 2%, respectively^29^].This is less than the approximate 10.3% reductions in raw volume seen for the mammillary bodies. The hippocampus was automatically segmented so one possibility is that the differences in degree of volume reduction could reflect differences in accuracy between manual and automatic segmentation. This was specifically addressed in a study by Arnold et al.^30^, where they found the absolute hippocampal volumes to be greater using Freesurfer segmentation compared to manual segmentation, however, the volumes using both methods correlated well in both healthy controls and patients with schizophrenia. Of note, both methods produced a reduction in hippocampal volume of approximately 2% in patients with schizophrenia, which is very similar to the mean volume loss of 3.58% found in the present cohort^30^. Alternatively, it may be that the mammillary bodies are more sensitive, and show greater atrophy than the hippocampus, at least in some individuals. Consistent with this, anterior thalamic nuclei volumes were reportedly affected in first-episode patients, at a point where no differences were observed in the hippocampus^31^. The mammillary bodies were also found to be significantly smaller in a genetic rodent model of schizophrenia, with mammillary volume correlating to psychotic symptomology, and this was found to be independent of hippocampal input^32^.

If the mammillary bodies are impacted in schizophrenia, it is likely that the major fibre tracts related to the mammillary bodies would also be affected. As previously mentioned, fornix integrity appears to be disrupted in schizophrenia^14^, although no study to date appears to have focused on the post-commissural fornix, the part of fornix that innervates the mammillary bodies^33^. There is also very little known about the mammillothalamic and mammillotegmental tracts in patients with schizophrenia as these tracts are very difficult to accurately reconstruct using standard imaging protocols^34^. However, the mammillotegmental and mammillothalamic tract are both smaller in an animal model of schizophrenia^32^, providing support for mammillary body-network changes.

As no background data were provided with this data set, it is not possible to determine the extent to which any volumetric changes may be exacerbated by lifestyle differences (e.g., increased alcohol consumption), co-occurring anorexia^5,35^, and/or treatment protocols or illness duration. However, the changes in mammillary body volume in younger patients, with both atrophy and swelling present across patients, suggest that while the mammillary bodies may show increased vulnerability to lifestyle and/or medication factors, their pathology is still likely to be a primary feature of schizophrenia. Consistent with this, the anterior thalamus, a brain region closely connected to the mammillary bodies, is also smaller in first-episode psychosis^31^. Furthermore, the mammillary bodies show abnormalities in animal models of schizophrenia, highlighting their vulnerability even without concomitant treatment and/or lifestyle differences^27,32^.

The lack of cognitive data for the current dataset means it is not possible to associate mammillary body volume with cognitive performance. However, previous studies have shown recollective memory performance significantly correlates with mammillary body volume, with poorer memory scores found in patients with smaller mammillary volumes^36–38^. Other studies have reported correlations between mammillary body volume and anxiety in patients with schizophrenia^12^.

Together, the findings from the present study provide further support for the involvement of the mammillary bodies in schizophrenia. The mammillary bodies are associated with both memory and anxiety^16^ and mammillary body pathology in schizophrenia could therefore contribute to both the cognitive impairments and changes in affect observed in this condition. A better understanding of the circumstances in which the mammillary bodies are most vulnerable, whether this is a genetic predisposition, treatment protocols, co-morbidities such as anorexia and or/lifestyle differences, would help identify ways to protect the mammillary bodies in schizophrenia and potentially help reduce accompanying symptomology.

## Supplementary information

Supplementary Information

## Data Availability

The scripts and data sets generated during the current study are available from the corresponding author on reasonable request.
