# Supplementary material for: Reduced mammillary body volume in individuals with a schizophrenia diagnosis: an analysis of the COBRE data set
Source: Schizophrenia (Heidelb). 2023 Aug 1;9(1):48. doi: 10.1038/s41537-023-00376-7 (PMC10394056; doi:10.1038/s41537-023-00376-7)
Supplement: Supplementary file 1 — Supplementary Information [file 41537_2023_376_MOESM1_ESM.pdf]

Supplementary Information

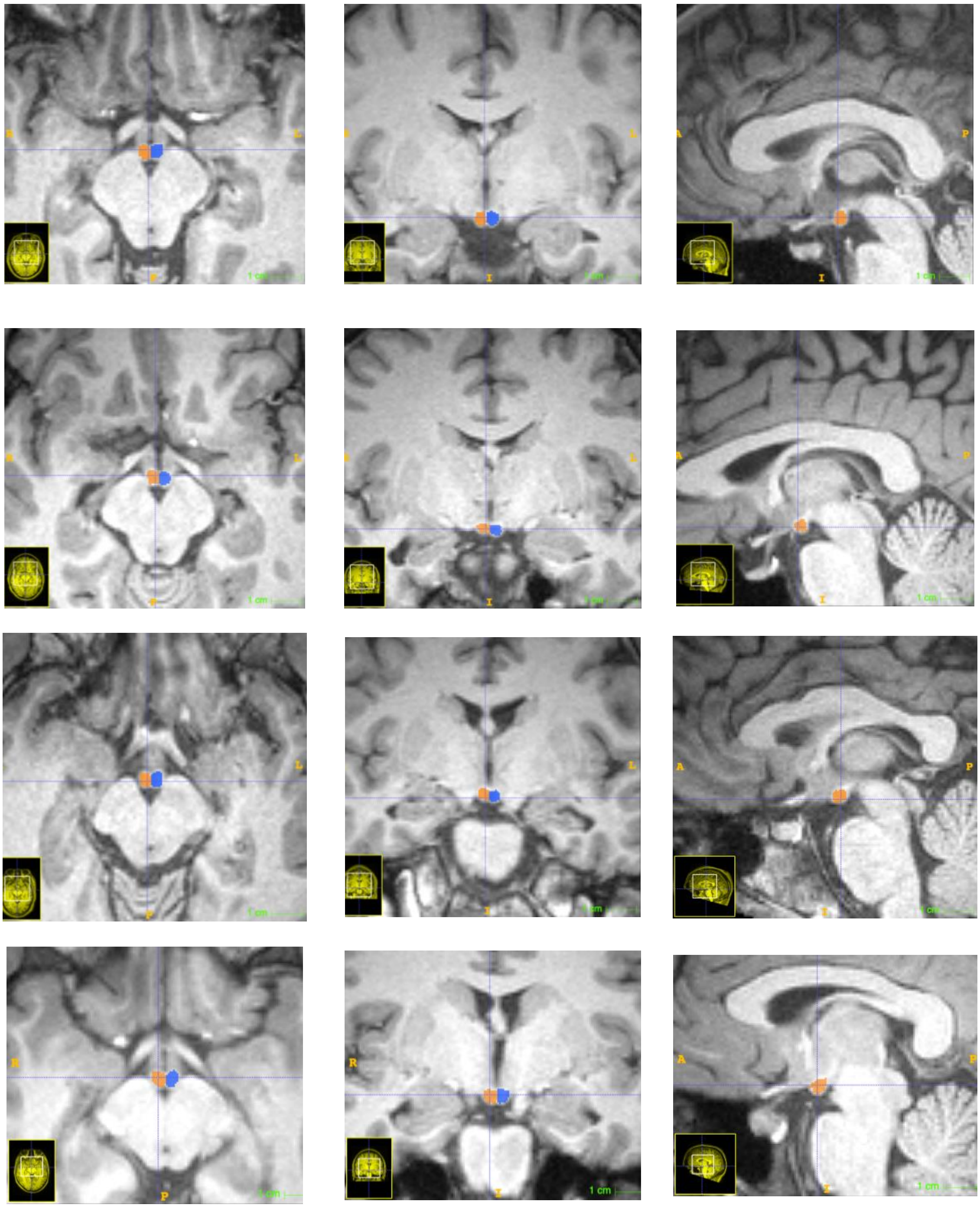

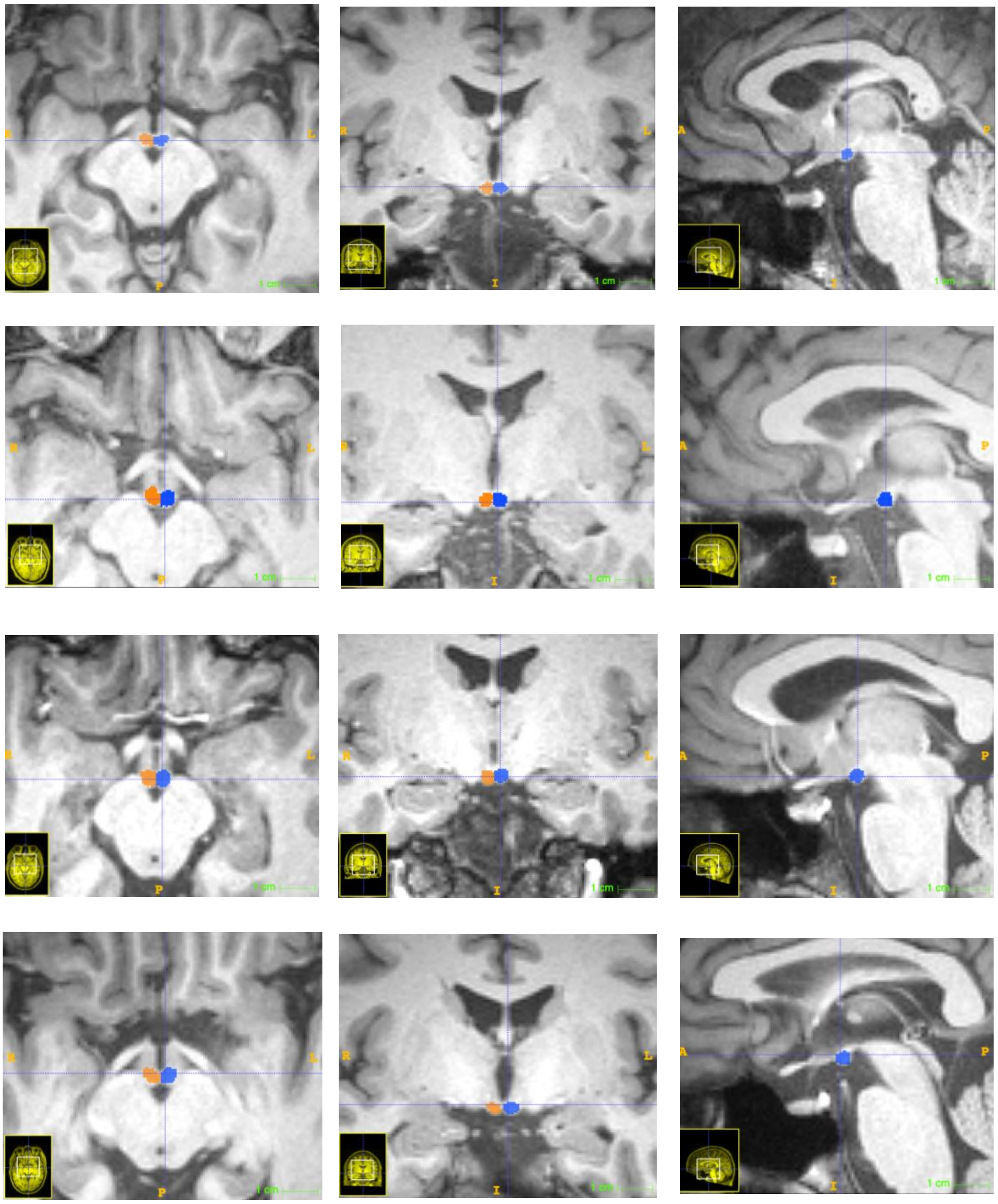

Figure S1: Examples of mammillary body segmentation using the ITK-SNAP software. Each image row represents axial, coronal and sagittal images from the same brain.

Table S1. Differences in ICV normalised volumes according to two methods. The top panel displays the statistics for ratio-normalised volumes and the bottom panel, for covariance-normalised volumes: median values, MAD – median absolute deviation, Bonferroni-adjusted *p* values, t values and effect sizes for Group comparisons in a generalized linear model. Significant differences have been highlighted in yellow. Abbreviations: MB_R: right mammillary body, MB_L: left mammillary body, MB_T: total mammillary bodies; HPC_L: left hippocampus, HPC_R: right hippocampus, HPC_T: total hippocampus; SUB_L: left subiculum, SUB_R: right subiculum, SUB_T: total subiculum; GM: total grey matter.

Method: Ratio	Median Control	MAD Control	Median Patient	MAD Patient	*p* value	t	Effect size
MB_R	2.54E-04	2.89E-05	1.99E-04	5.76E-05	2.50E-05	-4.91E+00	1.19
MB_T	5.13E-04	6.60E-05	4.18E-04	1.07E-04	5.21E-05	-4.74E+00	1.06
MB_L	2.64E-04	3.67E-05	2.26E-04	5.45E-05	3.53E-04	-4.27E+00	0.82
HPC_L	2.52E-03	1.76E-04	2.37E-03	1.98E-04	3.13E-02	-3.01E+00	0.81
HPC_T	5.10E-03	3.63E-04	4.75E-03	4.21E-04	7.05E-02	-2.73E+00	0.90
HPC_R	2.57E-03	1.95E-04	2.45E-03	2.38E-04	1.82E-01	-2.39E+00	0.55
GM	4.41E-01	2.97E-02	4.16E-01	3.61E-02	2.10E-01	-2.33E+00	0.74
SUB_L	3.22E-04	2.53E-05	3.12E-04	3.28E-05	2.14E-01	-2.33E+00	0.35
SUB_T	6.41E-04	4.67E-05	6.16E-04	5.55E-05	2.92E-01	-2.20E+00	0.48
SUB_R	3.16E-04	2.57E-05	3.06E-04	2.50E-05	6.01E-01	-1.90E+00	0.40

Method: Covariance	Median Control	MAD Control	Median Patient	MAD Patient	*p* value	t	Effect size
MB_R	2.61E+00	4.22E+00	-4.28E+00	7.72E+00	8.88E-06	-5.14E+00	1.11
MB_T	4.84E+00	9.58E+00	-6.46E+00	1.46E+01	1.38E-05	-5.04E+00	0.92
MB_L	2.88E+00	5.36E+00	-2.20E+00	7.63E+00	9.72E-05	-4.59E+00	0.77
HPC_L	8.89E+00	1.91E+01	-5.93E+00	2.51E+01	1.83E-03	-3.84E+00	0.66
HPC_T	1.94E+01	3.59E+01	-1.10E+00	5.91E+01	6.68E-03	-3.48E+00	0.62
HPC_R	8.15E+00	2.17E+01	-7.43E+00	3.12E+01	3.80E-02	-2.94E+00	0.58
SUB_L	8.07E-01	2.86E+00	-1.45E+00	3.96E+00	6.17E-02	-2.78E+00	0.65
SUB_T	1.75E+00	5.34E+00	-1.14E+00	6.85E+00	6.88E-02	-2.74E+00	0.47
GM	1.29E+03	3.32E+03	-1.08E+03	4.66E+03	7.96E-02	-2.69E+00	0.59
SUB_R	1.12E+00	2.51E+00	-4.47E-01	3.30E+00	1.93E-01	-2.37E+00	0.53

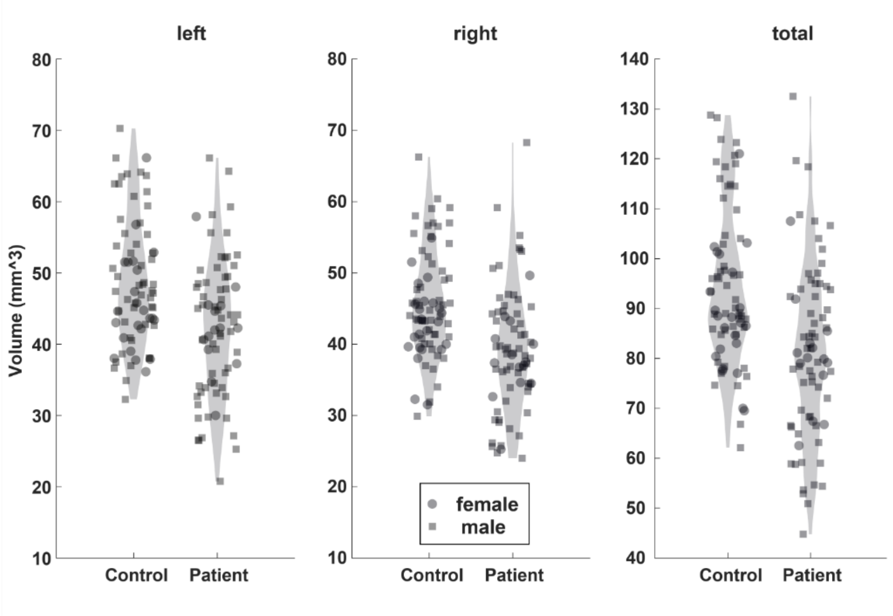

Figure S2. Scatter and violin plots of unadjusted mammillary body volumes. Circles – females, squares – males.

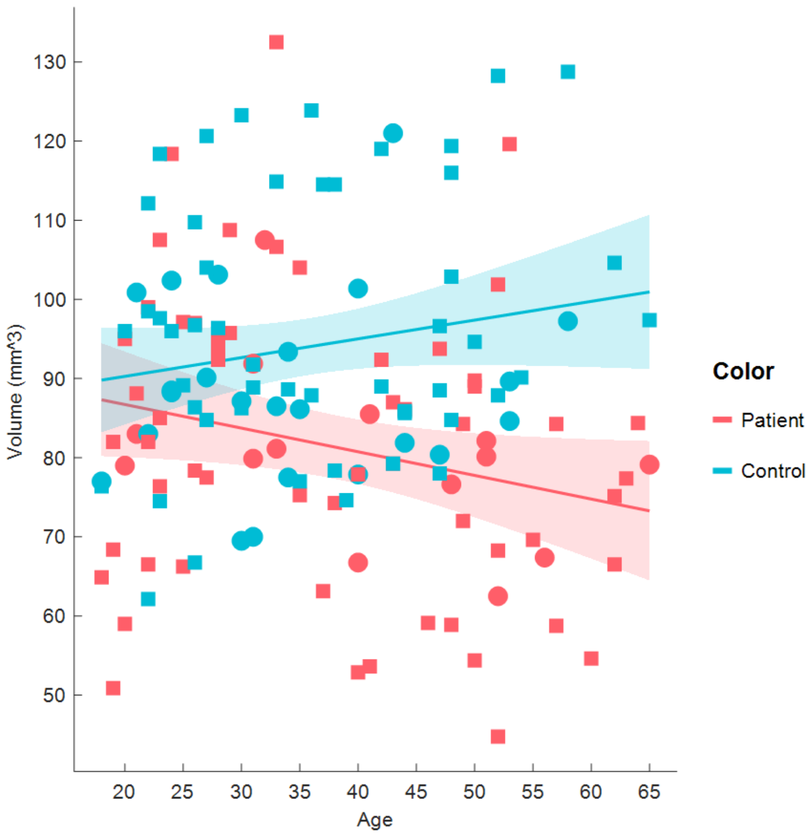

Figure S3. Correlation of unadjusted (total) mammillary body volumes with age in Controls and Patients. The lines are general linear model fits of the data. The shaded areas are the 95% confidence interval. Circles – females, squares – males.
